# Anorectal injury related to a personal watercraft: a case report and literature review

**DOI:** 10.1186/s40792-020-00993-9

**Published:** 2020-09-25

**Authors:** Kaoru Katano, Yuichiro Furutani, Chikashi Hiranuma, Masakazu Hattori, Kenji Doden, Yasuo Hashidume

**Affiliations:** Department of Surgery, Fukui Prefectural Hospital, 2-8-1, Yotsui, Fukui, Fukui 910-8526 Japan

**Keywords:** Anorectal injury, Personal watercraft, Strategy

## Abstract

**Background:**

Douche injury is a rare consequence of water recreation activities. Generally, this type of trauma occurs when people fall into the water in a sitting position during high-speed activities such as using a personal watercraft (PWC). Here, we report a rare case of anorectal injury caused by water jets from a PWC during sudden acceleration from rest.

**Case presentation:**

A 21-year-old male passenger on a PWC fell off backward from the rear seat when the craft suddenly accelerated. He fell into the water in a supine position with his legs open, and the water jets of the PWC struck his perineum directly. Thereafter, bleeding from the anus was seen, and he was transferred to our hospital. On physical examination, there was a deep laceration interrupting the external anal sphincter in the posterior rectal wall. Abdominal computed tomography revealed a full-layer perforation of the posterior rectal wall and leakage of feces into the extraperitoneal space, but intraperitoneal free air was not seen. Laparoscopic sigmoid loop colostomy and primary suturing of the sphincter and mucosa were performed. He did not have any complications and was discharged from our hospital 16 days after the surgery. His anal function was almost perfectly preserved, and his diverting colostomy was closed 4 months later.

**Conclusion:**

Anorectal injuries related to PWCs can occur not only while traveling at high speeds, but also when suddenly accelerating from rest. A diverting colostomy should be performed for this type of trauma. In these trauma cases, clinicians must suspect complex and life-threatening anorectal injuries early.

## Background

Douche injury, which occurs when a high-pressure water stream is injected into the orifices of the body [1], is a rare consequence of water recreation activities. Generally, this type of trauma occurs when people fall into the water in a sitting position during high-speed activities such as using a personal watercraft (PWC), water-skiing [1] or water slides [2]. Here, we report a rare case of anorectal injury caused by water jets from a PWC during sudden acceleration from rest.

## Case presentation

**Fig. 1:**
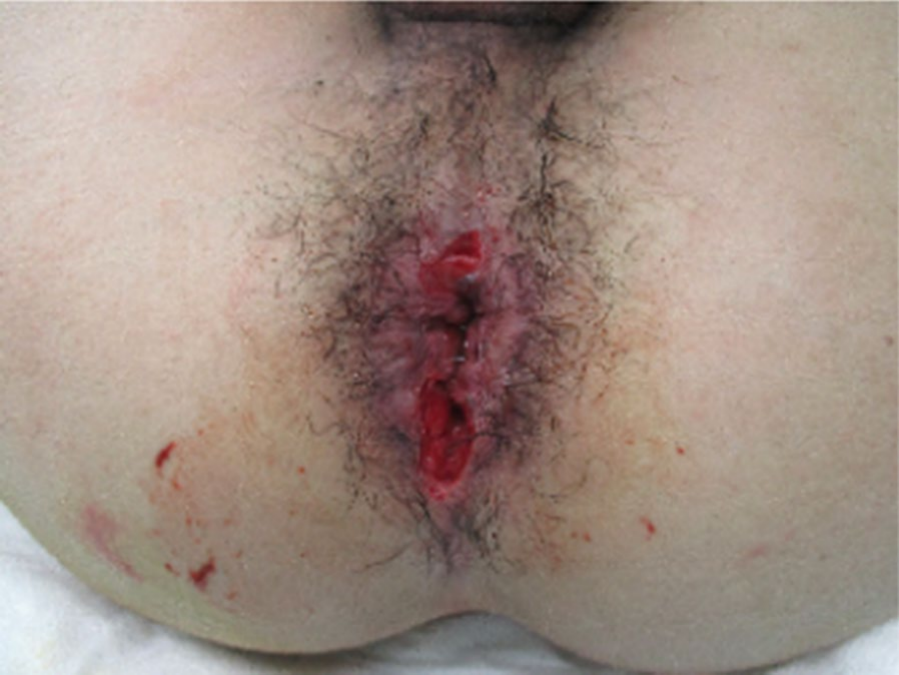
On perineal examination, there was 5-cm-deep laceration at the 0 and 6 o’clock locations in the perianal wall of the rectum

**Fig. 2:**
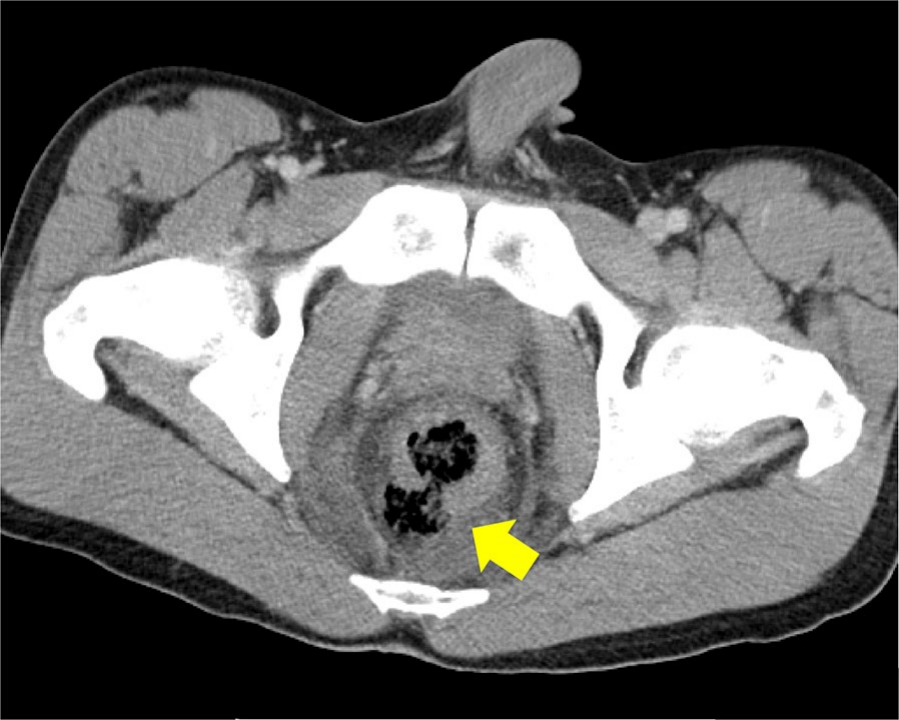
Abdominal CT scan revealed full-layer perforation in the posterior rectal wall and leakage of feces into the extraperitoneal space (yellow arrow)

**Fig. 3:**
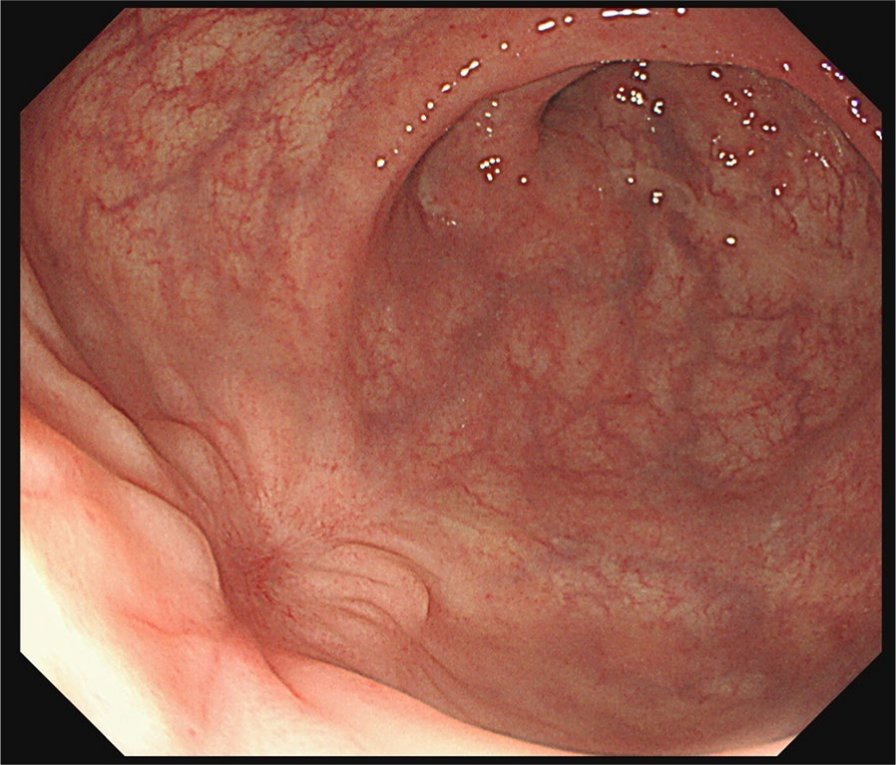
Colonoscopy confirmed that there were no abnormal findings such as stenosis in the sutured part of the rectal laceration

A 21-year-old man was riding on the back seat of a PWC as a passenger and was wearing a swimwear. After the craft suddenly accelerated, he fell into the water in a supine position with his legs open, and the water jets of the craft struck his perineum directly. Thereafter, bleeding from the anus was observed, and he was transferred to our hospital. His initial vital signs were stable. On physical examination, no significant findings in the abdomen were revealed. On perineal examination, there was a 5-cm-deep laceration at the 0 and 6 o’clock locations in the perianal wall of the rectum, and the lesion in the posterior rectal wall interrupted the external anal sphincter (Fig. 1). Digital examination of the anus demonstrated slight sphincter tone. Abdominal computed tomography (CT) revealed full-layer perforation in the posterior rectal wall and leakage of feces into the extraperitoneal space (Fig. 2), but intraperitoneal free air was not observed. Under general anesthesia, transanal primary repair of the perforation site was performed after lavage of the rectum. The sphincter and mucosa were closed separately with monofilament interrupted sutures. Thereafter, laparoscopic sigmoid loop colostomy with intraperitoneal drainage was performed. There was no dirty ascites in the abdominal cavity. He did not have any complications and was discharged from our hospital 16 days after the surgery. Three months after surgery, colonoscopy confirmed that there were no abnormal findings such as stenosis in the sutured part of the rectal laceration (Fig. 3). His anal function was almost perfectly preserved, and his diverting colostomy was closed 4 months later.

## Discussion

**Fig. 4:**
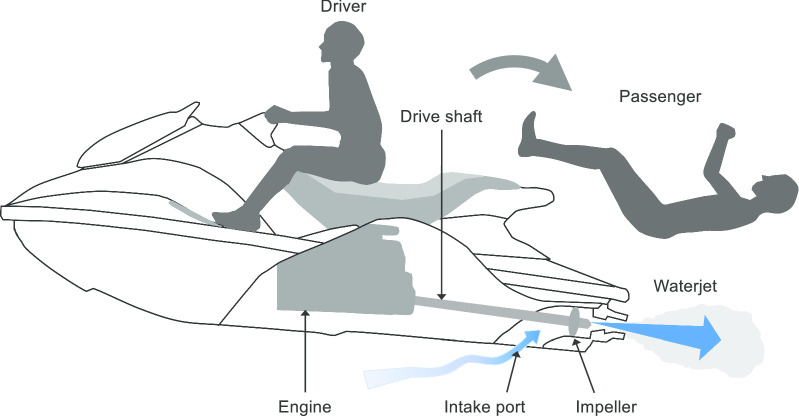
A PWC has a system in which the impeller is rotated by the engine, water is taken in from the intake port at the bottom of the hull, and the water flow is accelerated by a jet pump. If riders of PWCs fall backward off of the vehicle in a supine position with their legs splayed while the throttle is still activated, the water jets of the PWC will strike the perineum directly

The present case provided two important clinical suggestions. First, anorectal injuries related to PWC use can occur not only while traveling at high speeds, but also when suddenly accelerating from rest. PWC activities differ from other water activities in that the vehicle itself discharges water and creates a jet stream. A PWC has a system in which the impeller is rotated by the engine, water is taken in from the intake port at the bottom of the hull, and the water flow is accelerated by a jet pump (Fig. 4). If PWC riders fall backward off of the vehicle in a supine position with their legs splayed while the throttle is still activated, the water jets of the PWC will strike the perineum directly [3]. Experimental studies on pigs revealed that hydrostatic perforations of the colon occurred at an average pressure of 120 mmHg or greater, which is widely exceeded by the propulsion of water from a PWC. Therefore, the energy that these jets create is more than enough to create mucosal injuries and anorectal perforations [3, 4]. We found only 13 reports of this type of trauma with a search of Pubmed from 1998 to May 2020 using the search words “personal watercraft” and “jet ski” (Table 1) [3–14]. As shown in Table 1, in three cases, including the present case, patients were injured during sudden acceleration not during high-speed driving. This could be explained by the feature that a PWC itself creates a jet stream behind the vehicle.

**Table 1:** Reported cases of anorectal injuries related to PWC use

Reference	Year	Age/sex	Driver or passenger	Clothing	Situation/trigger	Injury	Sphincter injury	Treatment	Outcome
[4]	1998	14/F	Passenger	NA	Turbulence	Laceration in the rectum Dissection of the bowel wall Perforation of the recto-sigmoid colon	−	Resection Sigmoid end colostomy	Alive
[5]	1999	15/F	Passenger	Swimwear	Sudden acceleration from rest	Perianal laceration 1 * 8-cm perforation of the rectal wall	+	Primary closure Sigmoid loop colostomy	Alive, colostomy closure (5 months later)
[6]	1999	30/F	Passenger	Swimwear	NA	4-cm laceration in the rectal wall	−	Sigmoid loop colostomy	Alive, colostomy closure (12 weeks later)
[3]	2003	16/M	Passenger	NA	NA	5-cm laceration in the wall of the sigmoid colon Perforation of the rectal wall 2-cm linear wound of the anus	−	Resection Primary anastomosis	Dead
[7]	2004	26/F	Passenger	NA	Traveling at high speeds	5 * 2.5-cm laceration in the rectal wall 13-cm laceration in the rectal wall communicating with the vagina 3-cm laceration in the vaginal fornix	−	Primary closure Sigmoid end colostomy	Alive, colostomy closure (12 weeks later)
[8]	2007	28/F	Passenger	NA	Traveling at high speeds	Perforation of the anorectal wall 5-cm serosal damage to the sigmoid colon	+	Primary closure Sigmoid loop colostomy	Alive, colostomy closure (3 months later)
[9]	2007	15/F	Passenger	NA	Sudden acceleration	Deep perineal laceration Complete anodermal dissection	+	Primary closure Sigmoid loop colostomy	Alive, colostomy closure (timing unknown)
[9]	2007	19/F	Passenger	NA	NA	3-cm laceration in the rectal wall Laceration in the rectal wall Perforation of the rectosigmoid colon	+	Sigmoid end colostomy	Alive, schedule for colostomy closure
[10]	2007	34/M	Passenger	NA	NA	Perforation of the rectal wall	−	Sigmoid loop colostomy	Alive, colostomy closure (10 weeks later)
[11]	2009	28/M	Passenger	Swimwear	Jumping the waves	Long superficial wound of the perineum 3-cm mucosal damage to the rectal wall	−	Sigmoid loop colostomy	Alive, colostomy closure (20 weeks later)
[12]	2011	14/F	Passenger	Swimwear	Traveling at high speeds	Perforation of the rectal wall	−	Primary closure End colostomy	Alive, colostomy closure (4 months later)
[13]	2012	20/M	Passenger	NA	Traveling at high speeds	5-cm perforation of the rectal wall	−	Laparoscopic end colostomy	Alive, colostomy closure (6 months later)
[14]	2014	27/F	NA	NA	Traveling at high speeds	5-cm laceration of the rectal wall Perforation of the rectum	+	Primary closure End colostomy	Alive, colostomy closure (3 months later)
Our case	2020	21/M	Passenger	Swimwear	Sudden acceleration from rest	Deep laceration in the rectal wall Perforation of the rectal wall	+	Primary closure Laparoscopic sigmoid loop colostomy	Alive, colostomy closure (4 months later)

*NA *not available

The second clinical suggestion is that a diverting colostomy should be performed for this type of trauma. Previous studies have reported that primary repair or resection and primary anastomosis should be performed for colonic and intraperitoneal rectal injuries when there is no major physiologic abnormality [15]. For extraperitoneal rectal injuries, primary repair with or without diversion is the mainstay of treatment, but colostomy alone without repair may be considered for injuries that are technically difficult to access [15, 16]. However, in cases of anorectal injuries related to PWC use, as shown in Table 1, the lesions are complex and can occur in multiple locations and in a widespread area. We believe that patients who have sustained this trauma should not be treated with the above strategy. In addition, preoperative and intraoperative accurate and rapid assessments of the lesions associated with this trauma may be difficult to accomplish in emergency situations because the location and severity of lesions vary according to each case. Although all but one of the reported patients underwent a diverting colostomy and had a good prognosis, one case of death was reported. The poor prognosis in this case was caused by multisystem failure after primary closure of the injured wall of the sigmoid colon without colostomy with missed perforation of the anterior rectal wall [3]. We must be aware that primary repair or resection and primary anastomosis without colostomy with a missed lesion may cause mortality [3]. In the present case, we could have repaired the anorectal laceration transanally, but it was difficult to assess the damage to the upper part of the rectum and sigmoid colon carefully before or during the operation. Our patient underwent a colostomy due to concerns about sphincter dysfunction, wound contamination, and other damage at the proximal site and showed a good prognosis.

From a review of the literature, we summarized important points when treating this type of injury (Table 2). Vaginal injury caused by a high-pressure water douche has also been reported and may occur in association with anorectal injury [7, 17]. These cases often show arterial bleeding and hemorrhagic shock, and a hemostatic strategy such as vaginal packing [17] or hypogastric artery ligation [18] will be required. Therefore, primary treatment for this injury should focus on controlling bleeding and treating hemorrhagic shock [17]. If the patient's general condition is stable, preoperative and intraoperative adequate examinations are required to assess and repair the injuries. Perineal examination must include vaginal examination, and careful inspection for communication of rectal lacerations with the vagina is necessary in females [9]. In addition, several reports have described that rigid proctoscopy should be performed under general anesthesia [6, 9]. As shown in Table 1, perforation of the rectum or sigmoid colon is often observed in this type of injury. An abdominal CT scan is necessary to rule out bowel perforation and intraabdominal injuries. As mentioned above, a diverting colostomy is required for this type of injury. Primary repair, resection and drainage should be performed according to the location and severity of the lesions in each patient. As shown in Table 1, this type of trauma occurs more often in young people, and 6 cases showed anal sphincter injury. Suitable evaluation and recovery of sphincter function are very important for the quality of life of young patients. If there is dysfunction of the anal sphincter, proper repair and rehabilitation of the sphincter muscle are required. Furthermore, antibiotics should be given depending on the water source. The pathogens to consider in freshwater injuries include *Aeromonas* species, *Edwardsiella tarda*, and *Erysipelothrix rhusiopathiae*. For brackish or saltwater, additional coverage for *Vibrio* species should be considered [17].

**Table 2:** The treatment strategy for the douche injury related to a PWC

1. First, suspect perineal injuries due to mechanism of the douche injury
2. Stabilize vital signs (hemostasis, transcatheter arterial embolization, blood transfusion if necessary)
3. Perform necessary examinations (perineal visual examination/palpation, rectal examination, vaginal examination, contrast-enhanced torso CT, etc.)
4. Need to repair the anal sphincter and place a colostomy
5. Administer appropriate antibiotics estimated from the injured location (sea, lake, etc.)

Finally, as the other authors of similar literature reports have described, the prevention of accidents and injuries related to PWC use should be a primary goal for users [3, 6, 9]. None of the 14 cases reported that the victims had worn any specifically produced protective clothing, such as a wetsuit. Our patient was only wearing a swimwear, which could not provide protection against the high-pressure water jets that propelled the PWC. Therefore, wearing a wetsuit is recommended not only for drivers, but also for passengers. In addition, almost all the victims of this type of trauma were passengers in the 14 case reports. There are two reasons why these injuries occur in passengers rather than in drivers. First, compared with drivers, passengers have an increased risk of losing their balance during a ride. Because of the absence of a handlebar for maintaining stability as passengers, these individuals may not be ready to brace themselves for a sudden jolt or acceleration [9]. Safety education for both drivers and passengers should be considered. Second, passengers do not have an automatic shut-off switch. PWCs have an automatic shut-off switch with a cord attachment that stops the engine when the driver falls off, but no such cord is attached to passengers [9]. Therefore, the throttle can still be activated if the passenger falls off. Modifications to PWCs to provide the passenger with an automatic shut-off switch might reduce the chances of a fallen passenger being injured by the water jets.

## Conclusion

Anorectal injuries related to PWC use can occur not only while traveling at high speeds, but also when suddenly accelerating from rest. A diverting colostomy should be performed for this type of trauma. In addition, bleeding control, adequate perineal examination, repair or resection of damage sites, recovery of sphincter function, and suitable antibiotics should be performed if required. In this type of trauma, clinicians must suspect complex and life-threatening anorectal injuries early.

## Data Availability

All data generated during this study are included in this published article.

## Abbreviations

PWC: Personal watercraft
CT: Computed tomography
